# No Interaction between tDCS Current Strength and Baseline Performance: A Conceptual Replication

**DOI:** 10.3389/fnins.2017.00664

**Published:** 2017-12-01

**Authors:** Gemma Learmonth, Francesca Felisatti, Numaya Siriwardena, Matthew Checketts, Christopher S. Y. Benwell, Gesine Märker, Gregor Thut, Monika Harvey

**Affiliations:** ^1^Centre for Cognitive Neuroimaging, Institute of Neuroscience and Psychology, University of Glasgow, Glasgow, United Kingdom; ^2^School of Psychology, University of Glasgow, Glasgow, United Kingdom

**Keywords:** spatial attention, pseudoneglect, tDCS, plasticity, replication, bayesian analysis

## Abstract

Several recent studies have reported non-linear effects of transcranial direct current stimulation (tDCS), which has been attributed to an interaction between the stimulation parameters (e.g., current strength, duration) and the neural state of the cortex being stimulated (e.g., indexed by baseline performance ability, age) (see Fertonani and Miniussi, 2016). We have recently described one such non-linear interaction between current strength and baseline performance on a visuospatial attention (*landmark*) task (Benwell et al., 2015). In this previous study, we induced a small overall rightward shift of spatial attention across 38 participants using bi-hemispheric tDCS applied for 20 min (concurrent left posterior parietal (P5) anode and right posterior parietal (P6) cathode) relative to a sham protocol. Importantly, this shift in bias was driven by a state-dependent interaction between current intensity and the discrimination sensitivity of the participant at baseline (pre-stimulation) for the landmark task. Individuals with high discrimination sensitivity (HDS) shifted rightward in response to *low-* (1 mA) but not *high*-intensity (2 mA) tDCS, whereas individuals with low discrimination sensitivity (LDS) shifted rightward with *high-* but not *low*-intensity stimulation. However, in Benwell et al. (2015) current strength was applied as a between-groups factor, where half of the participants received 1 mA and half received 2 mA tDCS, thus we were unable to compare high and low-intensity tDCS directly *within* each individual. Here we aimed to replicate these findings using a within-group design. Thirty young adults received 15 min of 1 and 2 mA tDCS, and a sham protocol, each on different days, to test the concept of an interaction between baseline performance and current strength. We found no overall rightward shift of spatial attention with either current strength, and no interaction between performance and current strength. These results provide further evidence of low replicability of non-invasive brain stimulation protocols, and the need for further attempts to replicate the key experimental findings within this field.

## Introduction

Transcranial direct current stimulation (tDCS) has been used as a non-invasive method of modulating neuronal excitability in both healthy and clinical groups for over 15 years (Nitsche and Paulus, 2000). However, its ability to *reliably* modulate activity and behavior has recently been questioned. High variability of response to tDCS is often reported, both inter-individually (when the same protocol is administered to different people) and intra-individually (when a protocol is administered to the same people on different occasions; Krause and Kadosh, 2014; López-Alonso et al., 2014; Wiethoff et al., 2014; Chew et al., 2015; Dyke et al., 2016; Horvath et al., 2016b; Tremblay et al., 2016). Additionally, prior positive results have failed to replicate (Koenigs et al., 2009; Costa et al., 2015; Horvath et al., 2016a; Vannorsdall et al., 2016) and a growing body of null results has led to uncertainty in regarding tDCS as an effective method of neuromodulation (Minarik et al., 2015; de Hollander et al., 2016; Mungee et al., 2016; Tremblay et al., 2016; Westwood et al., 2016; Nilsson et al., 2017; Seyed Majidi et al., 2017; Verhage et al., 2017). A series of meta-analyses of the tDCS literature has found no strong evidence of efficacy within any cognitive domain probed (Horvath et al., 2015a), no benefits of single-session tDCS in healthy adults (Horvath et al., 2015b) and only a small reliable effect of tDCS in the modulation of motor-evoked potentials (Horvath et al., 2015c, although see Horvath et al., 2016a for a failed replication of this small effect using behavioral outcome measures). These points of contention, coupled with a high probability of publication bias in favor of positive study outcomes, have generated debate within the electrical stimulation research community regarding the overall efficacy of this technique (see Antal et al., 2015; Horvath, 2015; Price and Hamilton, 2015).

Yet, our understanding of the tDCS mechanism has evolved in recent years, with its effects now considered to be at least partially *non-linear* and *state-dependent* (see Fertonani and Miniussi, 2016 for review). Rather than evoking a consistent anode-excitation and cathode-inhibition response, it is likely instead that the stimulation parameters selected (e.g., current strength, duration, electrode size, online vs. offline application) dynamically interact with the endogenous characteristics of the individual who is receiving stimulation (e.g., age, gender, expertise, coupled with transient fluctuations in alertness, neurochemistry etc.). Response to tDCS is further influenced by the characteristics of the task under investigation (e.g. task type, difficulty and the various cognitive strategies employed by participants; Dockery et al., 2009; Stagg et al., 2011; Berryhill and Jones, 2012; Tseng et al., 2012; Batsikadze et al., 2013; Hsu et al., 2014, 2016; Bortoletto et al., 2015; Cabral et al., 2015; Gill et al., 2015; Learmonth et al., 2015; Gözenman and Berryhill, 2016; Shen et al., 2016). This variability may be further exacerbated in clinical populations (e.g., stroke), where patients exhibit a larger degree of neural heterogeneity relative to healthy individuals. Thus, although tDCS may exert a weak effect *overall*, resulting in the negative outcomes reported in the aforementioned meta-analyses, the efficacy and reliability of this technique might be improved by identifying grouping variables that *do* reliably generate behavioral effects, and the application of individualized stimulation parameters based on these characteristics.

Our research group has recently reported a significant non-linear effect of tDCS in modulating visuospatial attention in healthy young adults (Benwell et al., 2015). Young adults tend to over-represent the left side of space relative to the right, giving rise to a small leftward bias (*pseudoneglect*, Bowers and Heilman, 1980) that can be quantified using the line bisection and “landmark” tasks (Milner et al., 1993). Pseudoneglect is thought to arise due to a right parieto-occipital dominance for spatial attention, resulting in an imbalanced allocation of spatial attention favoring the left hemispace (Fink et al., 2000a,b; Fink et al., 2001; Foxe et al., 2003; Çiçek et al., 2009; Thiebaut de Schotten et al., 2011; Benwell et al., 2014; Longo et al., 2015). In Benwell et al. (2015), we aimed to induce directional shifts of spatial attention by increasing neural excitability in the parietal cortex in one hemisphere, whilst simultaneously reducing excitability of the homologous region in the opposite hemisphere. To this effect, we applied 20 min of dual-hemispheric tDCS to the left and right posterior parietal cortices (P5/P6) across three within-subject sessions (i) P5-anode/P6-cathode (LA/RC), (ii) P5-cathode/P6-anode (LC/RA), and (iii) sham (30 s of counterbalanced LA/RC or LC/RA). Secondly, we hypothesized that low- and high-intensity tDCS would differentially affect behavioral response, and therefore half of the participants received low- (1 mA) and half received high-intensity (2 mA) tDCS, in a between-groups manipulation. In line with previous work by Giglia et al. (2011), we successfully induced a small overall rightward shift of visuospatial attention relative to sham using the LA/RC montage, but we did not enhance the leftward pseudoneglect bias further using the reversed (LC/RA) montage. Importantly, there was a clear interaction between current strength and the endogenous baseline discrimination sensitivity of the participant, as indexed by the landmark task. For the LA/RC condition, individuals who performed the task with high precision exhibited a left-to-right spatial attention shift in response to 1 mA stimulation but not 2 mA, and those with low baseline discrimination sensitivity exhibited the same rightward shift in response to 2 mA but not 1mA. Thus, the behavioral response to tDCS was both non-linear (a different response to 1 and 2 mA) and state-dependent (a different response based on the performance of the participant).

Nonetheless, it is imperative that these “explanatory” grouping variables are also subjected to experimental replication to test their veracity and reliability. Here we aimed to conceptually replicate Benwell et al. (2015) to determine whether the previously-reported interaction between current strength and baseline discrimination sensitivity is a robust effect. Because a between-groups design was used previously (1 and 2 mA tDCS were applied to different participants) we were unable to assess whether the two tDCS intensities elicited *distinct* behavioral effects *within* each of the individuals tested. Thus, here we selected only the P5-anode/P6-cathode (LA/RC) montage, which showed a significant effect in both Benwell et al. (2015) and Giglia et al. (2011), and applied 1, 2 mA and sham tDCS in a *within-subjects design*. We expected to replicate the rightward visuospatial attention shift in individuals with *high discrimination sensitivity* (HDS) in response to low-current (1 mA), but not high-current (2 mA) stimulation. In contrast, individuals with *low discrimination sensitivity* (LDS) were expected to shift rightwards in response to 2 mA, but not 1 mA tDCS.

## Method

### Participants

Thirty right-handed young adults (16 female, mean age = 21.43, *SD* = 1.87, range = 19–25) were recruited. One male participant was excluded due to poor adherence to the task (baseline curve width >3 standard deviations above the group mean). All reported normal or corrected-to-normal vision and had no contraindications to tDCS (pacemaker, seizure etc., as per Rossi et al., 2009). The study was approved by the University of Glasgow College of Science and Engineering ethics committee and written, informed consent was obtained from each participant.

### tDCS

A direct current was applied using a battery-driven constant current stimulator (NeuroConn GmbH, Germany). Each experimental session involved a bi-parietal left-anode right-cathode (LA/RC) electrode montage, with the anode centered on P5 of the 10-20 International EEG system, and the cathode centered on P6. Three experimental conditions were administered in a single-blind, counterbalanced, within-subject design: (i) 1 mA tDCS for 15 min, (ii) 2 mA tDCS for 15 min, (iii) 1.5 mA tDCS for 30 s (sham protocol), all with an additional 30 s ramp-up and 30 s ramp-down period. One condition was administered per session, with ≥24 h between sessions per individual. Both carbon rubber electrodes measured 4 × 4 cm and were held in place using Ten20 conductive paste (Weaver & Co. Aurora, USA) to ensure sustained contact with the scalp. Electrode impedance was not formally assessed, but always measured <55 kΩ according to the maximum allowable limit of the tDCS device.

### Landmark task

Stimuli were presented using E-Prime 2.0 (Psychology Software Tools Inc., Pittsburgh, PA) via a Dell Precision T3400 PC and 19.5″ Sun Microsystems CRT monitor (with 1280 × 1024 pixel resolution and 100 Hz refresh rate). Viewing distance was fixed at 0.8 m using a chin rest.

**Figure 1:**
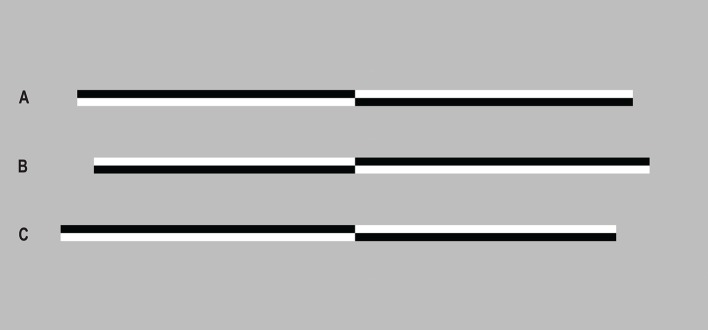
Landmark task stimuli. **(A)** Both sides are of equal length, **(B)** the left side is shorter than the right by 48 pixels and **(C)** the right side is shorter than the left by 48 pixels.

The landmark task (or “tachistoscopic line bisection,” McCourt and Jewell, 1999) is a computerized, two-alternative forced-choice version of the line bisection task. The stimuli were reproduced identically to those used in Benwell et al. (2015) (Figure 1). Horizontal lines of 100% Michelson contrast, measuring 800 × 14 pixels (0.24 × 0.04 m, 17.34 × 0.29° visual angle), were presented on a gray background (luminance = 179, hue = 160). Each line was transected vertically in the center of the screen at the same position as the fixation cross, and the length of the left and right sides of the line varied across trials. Seventeen different stimuli were created: 8 where the left side was longer than the right (by 48, 42, 36, 30, 24, 18, 12, or 6 pixels), 8 where the right was longer than the left (by 48, 42, 36, 30, 24, 18, 12, or 6 pixels) and 1 where both sides were of equal length. In half of the trials the line was shaded black in the lower right/upper left quadrants, and the upper right/lower left quadrants in the remaining trials. Each of the six landmark task blocks consisted of 272 trials (17 stimuli presented 16 times each in a random order), lasting ~7 min. A centrally located fixation cross appeared for 1500 ms, followed by a landmark stimulus for 150 ms. The fixation cross then reappeared until a response was given. Participants were instructed to press either the left (right index finger) or right (right middle finger) response keys, to indicate which side of the line they perceived to be shorter.

### Procedure

**Figure 2:**
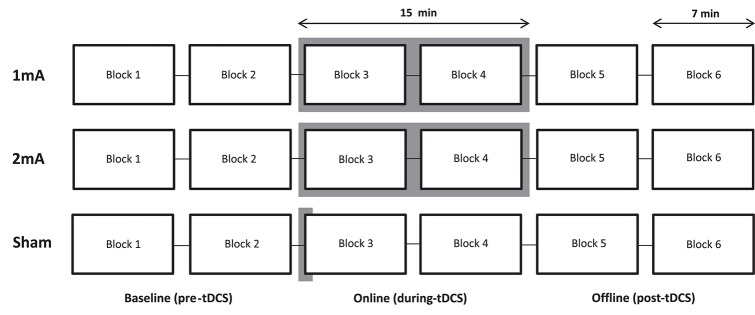
Experimental procedure. Each of the three conditions (1, 2 mA, and sham) was applied on a different day, ≥24 h apart, in a counterbalanced order across participants. The active stimulation period is highlighted.

Participants indicated their subjective alertness on a linear scale (0 = almost asleep, 100 = fully alert) at the beginning and end of each experimental session. The tDCS electrodes were applied to the scalp and the participant was seated with their midsagittal plane aligned with the computer screen. Blocks 1 and 2 of the landmark task were completed (*pre-tDCS*) to assess baseline task performance (Figure 2). Stimulation was then initiated and Blocks 3 and 4 of the landmark task commenced after the 30 s ramp-up period. A direct current was maintained throughout Blocks 3 and 4 (*during-tDCS*) in the two “active” (1 and 2 mA) conditions, and for the first 30 s of Block 3 in the sham condition, before ramp-down. Blocks 5 and 6 were then completed *post-tDCS*. A questionnaire was administered at the end of each session to assess the subjective experience of receiving tDCS (headache, tingling, itching, burning, pain. Score: 1 = “Not experienced at all,” 5 = “Experienced very strongly,” modified from Brunoni et al., 2011). To test for adequate subject blinding, subjects were invited to guess which of the three sessions involved sham tDCS at the end of their final session.

### Rationale for procedural differences between experiments

The study design of Benwell et al. (2015) was modified for the following reasons:

1. *Electrode montage:* Only the P5-a/P6-c (LA/RC) was selected for the present experiment, due to its efficacy in modulating spatial attention rightwards in Benwell et al. The P5-c/P6-a (LC/RA) montage did not enhance the leftward pseudoneglect bias previously, and was therefore not assessed further here.
2. *Within* vs. *between-groups design:* We aimed here to compare the effects of 1 and 2 mA tDCS directly within the same individuals, rather than across different subgroups as per Benwell et al. This was intended to determine whether tDCS-induced spatial attention shifts were specific to a particular current strength within individuals, or whether both current strengths elicited the same response within each subject. Additionally, within-subject designs are generally more powerful than between-groups designs, due to reduced variance across participants.
3. *Number and duration of blocks:* The number of experimental blocks was reduced from 10 in the prior study to six here, in order to improve the statistical power of the experiment. Each block was lengthened slightly in duration, with ~7 min per block, compared to 4–5 min previously. This also meant that more trials were included in the psychometric function fitting, providing a more accurate measure of spatial bias per block. Finally, 2 pre-tDCS baseline landmark task blocks were included rather than 1 used previously, to ensure a more stable measure of baseline performance.
4. *Duration of stimulation:* To ensure that Blocks 3 & 4 (now 7 min each) were performed entirely during stimulation, active stimulation was reduced from 20 to 15 min. This was deemed an appropriate modification because in Benwell et al. the peak online tDCS effects occurred well within this 15 min time window (see Figure 3 in Benwell et al., 2015). In addition, due to the shorter block duration in the previous experiment (4–5 min), individuals varied in the number of blocks that they performed online and offline (i.e., during and after tDCS), with the majority finishing active stimulation during Blocks 6 or 7. Thus, this modification strengthened the demarcation between online and offline tDCS periods here, as all participants finished tDCS during Block 4.
5. *Conductive medium:* Ten20 conductive paste was used to affix the electrodes to the scalp in the present experiment, compared to saline-soaked sponges used previously. This improved the contact between the electrodes and the scalp, and prevented uncontrolled distribution of the current via excess saline solution.
6. *Performance group allocation:* In Benwell et al. the participants were assigned to performance groups based on their mean Block 1 curve width across the three sessions (LA/RC, LC/RA & sham). The mean curve width was calculated separately for the 1 and 2 mA groups, and participants were assigned into four subgroups accordingly (1 mA HDS, 1 mA LDS, 2 mA HDS, 2 mA LDS). Here, the curve width was first averaged across Blocks 1&2, then averaged across the three sessions (1, 2 mA & sham). Participants were assigned to the four subgroups based on the *median* curve width, to ensure that similar numbers were allocated to each group.
7. *Viewing distance:* Due to laboratory restrictions, participants were seated 0.8 m from the computer screen during the landmark task, rather than 0.7 m as per the prior study.
8. *Sham current strength:* 1.5 mA was applied here, compared to 1 or 2 mA used previously, depending on current strength group allocation.

### Analyses

#### Landmark task

As per Benwell et al. (2015) the percentage of trials where the left side of the horizontal line was perceived to be shorter was calculated, separately for each of the 17 different stimuli. Psychometric functions were then fitted for each landmark task block, for each individual, using a cumulative logistic function:

$$\begin{array}{l} f\left(\mu ,\;x,\;s\right)=1/\left(1+\exp\left(\frac{x-\mu }{s}\right)\right) \end{array}$$

where μ is the point on the x-axis that corresponds to 50% left-shorter and 50% right-shorter response rate (i.e., the position along the horizontal landmark line that corresponds to where the individual perceived both sides of the line to be of equal length), *x* represents each of the 17 stimuli tested and *s* is the psychometric curve width. The point of subjective equality (PSE) and curve widths were extracted: the PSE is used to quantify spatial bias and provides a measure of the subjective midpoint of the landmark lines for each block, whereas the curve width estimates the precision of these judgements. High precision is indicated by a small (narrow) curve width and large (wide) values indicate low task precision.

#### Performance group allocation

Fifteen individuals whose mean baseline curve width was narrower than the median curve width of 6.31 were allocated to the HDS performance group, and 14 with a curve width wider than the median were allocated to the LDS group. The participant whose curve width acted as the group median value was allocated to the HDS group, given that one LDS participant was excluded due to very poor discrimination sensitivity prior to analysis.

## Results

The full dataset for this study is available at https://osf.io/ydw4u/.

### Subjective alertness questionnaire

Alertness generally reduced throughout the experiment (3 × 2 ANOVA: *tDCS-intensity (1ma, 2mA, sham)* × *time (pre, post):* effect of time [*F*_(2, 26)_ = 46.73, *p* < 0.001)] but there was no difference between the three stimulation conditions. There was no interaction between tDCS condition and time, suggesting that general alertness did not reduce (or increase) in response to one tDCS intensity relative to the other.

### tDCS sensory side-effects

A series of Friedman's tests to compare the severity of each of the five sensory side-effects (headache, tingling, itching, burning, and pain) found no difference in ratings between the three stimulation conditions (min *p* = 0.25). Thirteen participants (44.8%) correctly guessed which of their three conditions involved the sham protocol, compared to 19 (50%) in Benwell et al., both of which are above the 33% chance level. Guess accuracy did not differ between performance groups [HDS vs. LDS: $\chi_{\left(1,\;29\right)}^{2}$ = 0.29, *p* = 0.59].

### Baseline landmark task performance

**Figure 3:**
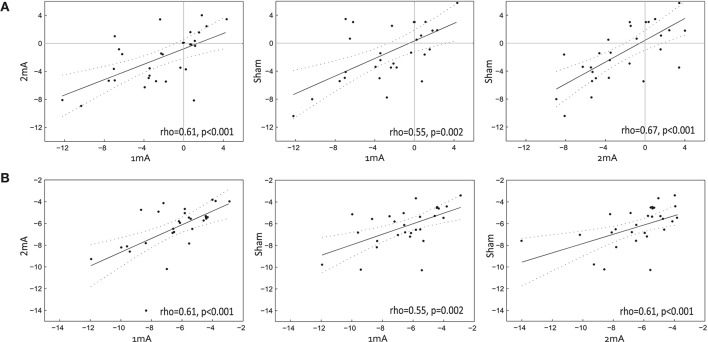
Baseline performance. The baseline (mean of Blocks 1&2) PSE correlations across the three tDCS conditions are shown in **(A)**, where the axis values represent the number of pixels relative to veridical horizontal midpoint. Baseline curve width correlations are shown in **(B)**, where the axis values represent the number of pixels. The slope and 95% confidence intervals are overlaid.

To ensure that each group exhibited a leftward (pseudoneglect) bias at baseline, one sample *t*-tests were performed for the baseline PSE (mean of Blocks 1 & 2), separately for the 1, 2 mA, and sham sessions. There was a numerically leftward group-level bias at baseline for all three conditions (mean 1 mA = −2.57, 2 mA = −2.24, sham = −1.33 pixels relative to the veridical midpoint of the landmark line). However, biases were only significantly different from zero (i.e., no bias) for the 1 mA [*t*_(28)_ = −3.42, *p* = 0.002] and the 2 mA [*t*_(28)_ = −3.28, *p* = 0.003] conditions, but were not significantly left of center for the sham condition [*t*_(28)_ = −1.7, *p* = 0.1]. A repeated measures ANOVA did not highlight any significant differences in baseline PSEs across the three tDCS conditions [*F*_(2, 56)_ = 2.0, *p* = 0.15]. In line with Benwell et al. (2015), the baseline PSEs were correlated across the three sessions (Figure 3), indicating a stable leftward spatial bias across testing days: 1 vs. 2 mA [Spearman's rho: r_(29)_ = 0.61, *p* < 0.001], 1 mA vs. Sham [*r*_(29)_ = 0.55, *p* = 0.002], 2 mA vs. Sham [r_(29)_ = 0.67, *p* < 0.001]. Baseline curve widths were similar across the three tDCS conditions [*F*_(2, 56)_ = 0.25, *p* = 0.78] and were also correlated across testing days, indicating a stability of task precision across sessions: 1 vs. 2 mA: r_(29)_ = 0.61, *p* < 0.001, 1 mA vs. Sham: r_(29)_ = 0.55, *p* = 0.002, 2 mA vs. Sham: r_(29)_ = 0.61, *p* < 0.001.

### Overall tDCS effects

#### Spatial bias (PSE)

**Figure 4:**
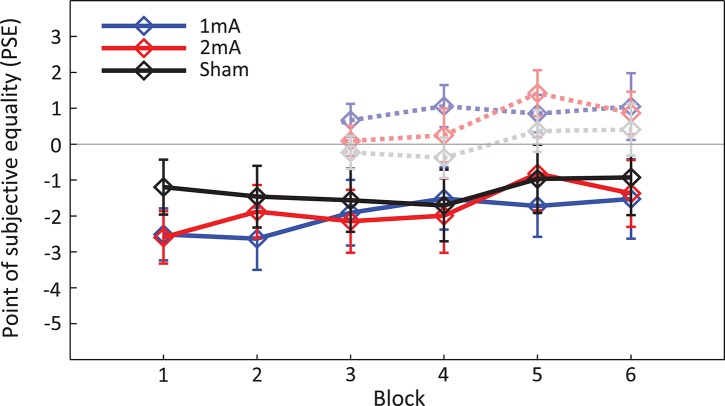
Grand average spatial attention bias (PSE). Solid lines represent the spatial bias (PSE) for Blocks 1–6 across all participants (± standard error of the mean). Negative values indicate a left bias and positive values a right bias. Transparent lines represent the baseline-normalized spatial bias shift for each block.

A full-factorial 3 × 6 (*tDCS-intensity* x *Block*) ANOVA performed on the spatial attention bias (PSE; Figure 4) measurements found no overall effect of tDCS intensity [*F*_(2, 56)_ = 0.55, *p* = 0.58, η*p*2 = 0.019], no PSE shifts related to experimental block [*F*_(5, 140)_ = 1.58, *p* = 0.17, η*p*2 = 0.05] and no intensity x block interaction [*F*_(10, 280)_ = 0.86, *p* = 0.57, η*p*2 = 0.03]. As per the analysis performed in Benwell et al. (2015), the data were then *sham-normalized* (i.e., the PSEs obtained during the sham protocol were subtracted block-by-block from the PSEs obtained during each of the two active stimulation conditions, separately for each individual). These sham-normalized data were then *baseline-corrected* (i.e., the mean baseline PSE (Blocks 1&2, averaged) was subtracted block-by-block from Blocks 3-6, separately for each individual). These data were then subjected to a 2 × 4 ANOVA (*tDCS-intensity* × *Block*) which found no main effects of stimulation [*F*_(1, 28)_ = 0.47, *p* = 0.5, η*p*2 = 0.017] nor block [*F*_(1, 28)_ = 0.14, *p* = 0.94, η*p*2 = 0.005] and no stimulation x block interaction [*F*_(1, 28)_ = 1.34, *p* = 0.27, η*p*2 = 0.045]. A *t*-test identified no difference in PSE shifts between the 1 and 2 mA tDCS conditions during the *online* stimulation period (mean of Blocks 3-4) [*t*_(28)_ = 1.61, *p* = 0.12] relative to baseline, nor was there any difference in PSE shifts between the two tDCS intensities over the course of the overall experiment (*online* and *offline*: mean of Blocks 3–6) [*t*_(28)_ = 0.69, *p* = 0.5]. This is in contrast to Benwell et al. where we identified a small overall rightward shift of spatial bias in response to the LA/RC montage relative to sham [a one sample *t*-test compared to zero, collapsed over both current strengths *t*_(37)_ = 2.003, *p* = 0.052]. The same analysis performed on the present replication data, averaged over both current strengths, identified no overall rightward shift relative to sham in response to the same LA/RC montage *t*_(28)_ = 1.31, *p* = 0.2.

#### Task precision (curve width)

The same analysis was then applied to the curve widths. Most notably, the 3 × 6 (*tDCS-intensity* × *Block*) ANOVA found a general reduction in task precision over the course of the experiment [*F*_(5, 140)_ = 7.6, *p* < 0.001, η*p*2 = 0.21] but this did not differ between the three stimulation conditions [Intensity × Block: *F*_(2, 280)_ = 4.19, *p* = 0.94, η*p*2 = 0.015].

### Group performance split: mean baseline performance across experiment

#### Spatial bias (PSE)

**Figure 5:**
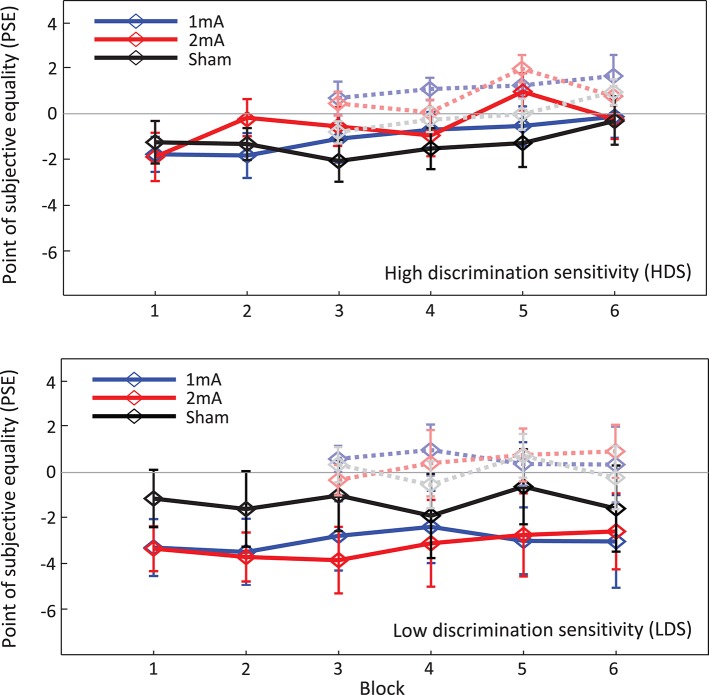
Grand average spatial attention bias (PSE), separated by performance group. Negative values indicate a leftward bias and positive values a rightward bias. Transparent lines represent the baseline-normalized shift of spatial bias for each block.

The analyses were performed again, but this time including *performance group* as a between-subjects factor (Figure 5). A full-factorial 3 × 6 × 2 ANOVA (*tDCS-intensity* × *Block* × *Performance*) on the PSE values once again found no effect of tDCS intensity [*F*_(2, 54)_ = 0.65, *p* = 0.53, η*p*2 = 0.02], no effect of Block [*F*_(5, 135)_ = 1.5, *p* = 0.19, η*p*2 = 0.5], no effects of performance group [*F*_(1, 27)_ = 1.26, *p* = 0.27, η*p*2 = 0.04] and no significant interactions. In contrast to Benwell et al. (2015), a 2 × 4 × 2 ANOVA performed on the *sham-removed* and *baseline corrected* PSE values found no interaction between tDCS intensity and baseline performance [*F*_(1, 27)_ = 0.089, *p* = 0.77, η*p*2 = 0.003] and no three-way interaction between tDCS intensity, performance and block [*F*_(3, 81)_ = 0.83, *p* = 0.48, η*p*2 = 0.03].

**Figure 6:**
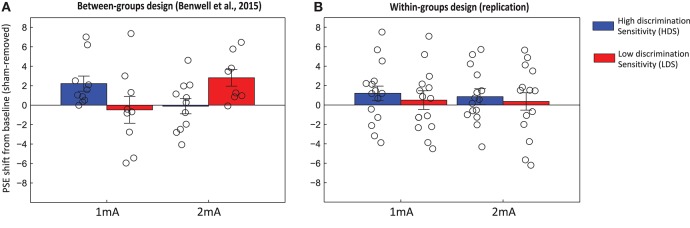
Mean shift of spatial bias (PSE) across Blocks 3–6, relative to baseline (Blocks 1 & 2), with Sham data extracted. Individual shifts for each participant are overlaid. Graph **(A)** represents the shifts observed in Benwell et al. (2015) using a between-groups design, and graph **(B)** shows the data from the present within-subjects design experiment.

Finally, we assessed the PSE shifts relative to baseline during the *online* stimulation period (i.e., mean of blocks 3–4), in which there were no significant effects of tDCS intensity [*F*_(1, 27)_ = 2.5, *p* = 0.13, η*p*2 = 0.085], performance group [*F*_(1, 27)_ = 0.25, *p* = 0.62, η*p*2 = 0.009] and no intensity x performance interaction [*F*_(1, 27)_ = 0.019, *p* = 0.89, η*p*2 = 0.001]. Likewise, the overall change in PSE during the experiment (*online* and *offline*: mean of Blocks 3–6; Figure 6) found, in contrast to Benwell et al. (2015), that there were no significant effects of either tDCS-intensity [*F*_(1, 27)_ = 0.44, *p* = 0.51, η*p*2 = 0.16], performance group [*F*_(1, 27)_ = 0.27, *p* = 0.61, η*p*2 = 0.01] and no interaction between tDCS intensity x performance [*F*_(1, 27)_ = 0.09, *p* = 0.77, η*p*2 = 0.003].

#### Task precision (curve width)

Due to the performance group allocation that was based on baseline curve width values, a 3 × 6 (*tDCS-intensity* × *Block*) ANOVA found a significant effect of performance group [*F*_(1, 27)_ = 18.41, *p* < 0.001, η*p*2 = 0.41], and also a Block × Performance interaction [*F*_(5, 27)_ = 3.70, *p* = 0.004, η*p*2 = 0.12]. A series of independent samples *t*-tests to directly compare the HDS and LDS groups, separately for each block (Blocks 3–6, baseline removed), found a between-group difference in Block 6 [*t*_(27)_ = 2.09, *p* = 0.046]. Thus, the HDS group maintained their baseline precision throughout the experiment, whereas the LDS group had further reduced precision by Block 6 compared to baseline.

### Consistency of response to tDCS

**Figure 7:**
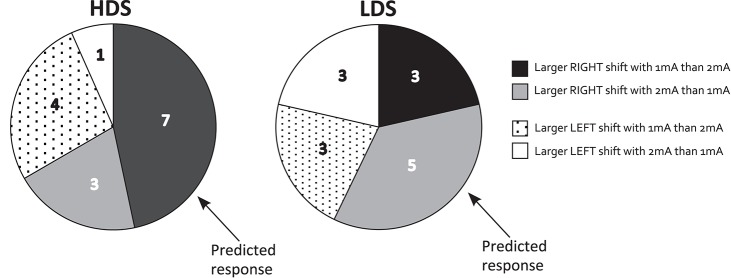
Distribution of behavioral response shown separately for high- and low discrimination sensitivity performance groups (HDS and LDS). The number of individuals in each performance group who exhibited left/right directional shifts of spatial attention in response to 1 and 2 mA tDCS (after baseline-correction and sham-extraction) are shown. Those conforming to the initial predictions as per Benwell et al. (2015) are highlighted.

Figure 7 illustrates the distribution of overall spatial attention shifts (mean of Blocks 3–6 relative to baseline, and sham data extracted) for each individual tested, separately for the 1 and 2 mA tDCS intensities and the LDS and HDS performance groups. Contrary to the results of Benwell et al. (2015), where *all* of the HDS individuals shifted rightward relative to sham with 1 mA, and all-but-one of the LDS performers shifted rightward with 2 mA, we found a high degree of variability of response to both tDCS intensities. We predicted that the majority of HDS individuals would shift rightward relative to sham with 1 mA but not, or to a lesser extent, with 2 mA. Although 10/15 HDS participants did shift rightward with 1 mA, only 7 (46.67% of total) had a larger rightward shift with 1 mA compared to 2 mA, as per our original hypothesis. Similarly, we predicted that LDS individuals would shift rightward with 2 mA but not 1 mA. We found that 8/14 of LDS participants shifted rightward with 2 mA, but only 5 (35.71% of total) shifted further right with 2 mA compared to 1 mA, as predicted.

### Group performance: separate performance split for each condition

**Figure 8:**
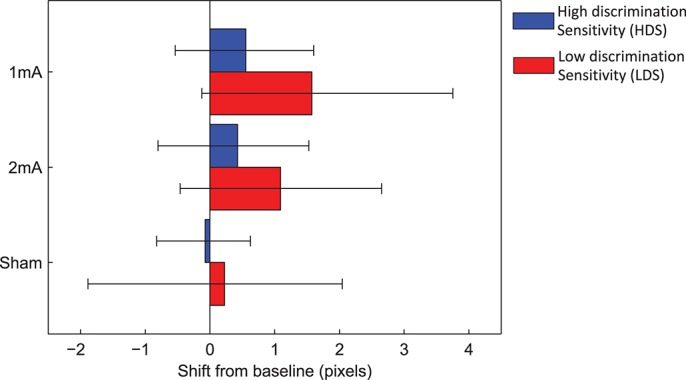
Mean spatial attention shifts, with performance split based on each condition separately. The mean curve width was calculated separately for each condition, and participants whose curve width fell above the mean were allocated to HDS, and below the mean to LDS. Ninety-five percentage confidence intervals are shown.

It is possible that by allocating participants into HDS and LDS performance groups based on average baseline performance across the three sessions, we may have failed to take into account between session variations in performance which could have contributed to our observed null effects of tDCS. Although 18/29 participants (62.1%) were allocated to the same group on all three occasions (11 HDS & 7 LDS), the remaining 11/29 (37.9%) were only allocated to the same group on 2 of 3 days. To assess this, we specifically probed spatial attention shifts in the 18 participants who were allocated consistently across the three sessions. A 2 × 2 ANOVA on the overall shifts of spatial bias from baseline (2 sham-extracted stimulation conditions × 2 performance groups) identified no overall effects of current strength [*F*_(1, 16)_ = 0.061, *p* = 0.81, η*p*2 = 0.004] nor performance group [*F*_(1, 16)_ = 0.007, *p* = 0.93, η*p*2 < 0.001] and no current strength x performance group interaction [*F*_(1, 16)_ = 0.49, *p* = 0.5, η*p*2 = 0.029]. Secondly, we then allocated participants to the HDS and LDS groups based on their mean baseline curve width for each condition separately (e.g., a participant may be a HDS performer in two of three conditions and LDS in the third). The mean spatial bias shift from baseline (Blocks 3–6 averaged) was then calculated and 95% confidence intervals were computed using 20,000 bootstrap permutations of the data. Figure 8 shows a substantial overlap of the confidence intervals for each stimulation condition and performance group, supporting the overall conclusion of no interaction between baseline performance and current strength.

### Bayesian comparison of studies 1&2

We then formally compared the outcomes of Studies 1 & 2 using Bayesian analysis. Based on Dienes (2014), this method takes the effect size of Study 1 into account and assesses whether a replication study is able to provide support for the null hypothesis (i.e., no evidence for a tDCS current and baseline performance interaction: a failed replication, where *B* < 1/3), the alternative hypothesis (i.e., evidence for an interaction: a successful replication, where *B* > 3) or whether the replication study is insensitive to support either hypothesis (where *B* >1/3 and <3).

#### Current strength × baseline performance interaction

In Benwell et al. we identified an interaction between baseline performance and current strength, where *F*_(1, 34)_ = 8.465, *p* = 0.006. For Study 1 the difference in bias shifts between the good and poor performance groups (in pixels) was calculated, separately for the 1 and 2 mA conditions (1 mA HDS = 2.22, 2 mA HDS = −0.11, 1 mA vs. 2 mA difference = 2.33; 1 mA LDS = −0.48, 2 mA LDS = 2.82, 1 mA vs. 2 mA difference = −3.30). The difference of these two differences was then calculated (5.63 pixels). The same method was used for Study 2: 1 mA HDS = 1.21, 2 mA HDS = 0.85, 1 mA vs. 2 mA difference = 0.36; 1 mA LDS = 0.5, 2 mA LDS = 0.36, 1 mA vs. 2 mA difference = 0.14; difference of differences = 0.219 pixels. The standard error of Study 2 was 0.735 (based on the current × performance interaction of *F* = 0.089 and corrected to 0.76 due to degrees of freedom of <30, as per Dienes, 2014). A one-tailed test performed via Dienes (2008; http://www.lifesci.sussex.ac.uk/home/Zoltan_Dienes/inference/bayes_factor.swf) estimated a Bayes factor of 0.17, indicating a failed replication of the current strength × baseline performance interaction from Benwell et al. (2015).

#### Overall rightward shift in response to LA/RC stimulation

In Benwell et al., there was an overall rightward shift of 1.03 pixels for the LA/RC montage relative to sham [one sample *t*-test against zero: *t*_(37)_ = 2.003, *p* = 0.052], but not the opposite (RA/LC) montage. In this replication study we identified a mean overall rightward shift of 0.74 pixels (SE = 0.57, corrected to 0.58), producing a Bayes factor of 1.58. The present data is therefore insensitive to enable a distinction between the theory and the null hypothesis for this secondary question.

## Discussion

We aimed here to conceptually replicate our recent study (Benwell et al., 2015) in which we reported a significant non-linear effect of tDCS that was dependent on both current intensity (1 vs. 2 mA) and the baseline state of the individual (high- vs. low-discrimination sensitivity on the landmark task). The experimental design was modified in order to allow a direct comparison of the two current intensities *within* individuals rather than *between* groups. As per our previous results, we predicted that individuals with high baseline task precision (HDS) on the landmark task would exhibit a left-to-right shift of spatial attention in response to only low-dose (1 mA) P5-anodal/P6-cathodal tDCS, whereas those with low baseline precision (LDS) would exhibit the same rightward attention shift in response to only high-dose (2 mA) stimulation. In contrast, we found no overall effects of bi-parietal tDCS on spatial attention bias relative to sham, and in particular, no evidence of an interaction between tDCS intensity and baseline task performance. These results provide further evidence of the poor replicability of positive tDCS findings (Koenigs et al., 2009; Costa et al., 2015; Horvath et al., 2016a; Vannorsdall et al., 2016) and the importance of selecting an appropriate experimental design to validate these effects.

### Inter-individual variability

In contrast to both Benwell et al. (2015) and Giglia et al. (2011) we did not succeed here in replicating the overall rightward shift of spatial attention bias with a bi-parietal left P5 anode/right P6 cathode montage. Although there was indeed a small *mean* rightward shift relative to the sham protocol in response to both stimulation intensities, and for both performance groups (Figure 6), these shifts failed to reach significance. The Bayesian analysis of this overall rightward shift, comparing the two studies, indicates that the replication data is unfortunately insensitive to allow us to distinguish between the presence or absence of an effect (BF = 1.58), although eliciting an overall stimulation effect was not the main aim of this study. In fact, there was a high inter-individual variability of response during both tDCS protocols in this dataset which was not present in the previous study (Krause and Kadosh, 2014; López-Alonso et al., 2014; Wiethoff et al., 2014; Chew et al., 2015; Dyke et al., 2016; Horvath et al., 2016b; Tremblay et al., 2016). In Benwell et al. (Figure 6A) *all* of the HDS individuals who received 1 mA shifted rightward relative to sham, and similarly, *all* of the LDS individuals shifted rightward with 2 mA, giving rise to a convincing performance x intensity interaction. There was no such group-level consistency in the present data (Figure 6B).

Why might these differences in effects have arisen between the two datasets? One possibility is statistical error: a Type I error in Benwell et al., based on a relatively small sample size (four groups of 8–11 individuals) might have generated this statistically significant effect by chance. Indeed, our replication sample of 29 individuals here is again (admittedly) small, yet the Bayesian comparison of both studies for the interaction effect suggests that the data in Study 2 are adequately sensitive to interpret this as a failed replication (BF = 0.17). Indeed, these sample sizes are consistent with those adopted in the current non-invasive brain stimulation literature and serve to highlight the need for robust replication of outcomes with appropriately large sample sizes (Minarik et al., 2016).

### Importance of experimental design

Only 7/15 of our HDS performers exhibited a larger rightward spatial bias shift with 1 mA than with 2 mA, and 5/14 LDS individuals exhibited a larger rightward shift with 2 mA compared to 1 mA, as per our original predictions (Figure 7). Due to the between-group design of Benwell et al., we were previously unable to probe this response across the two stimulation intensities. Therefore, in addition to highlighting the need for robust replication of non-invasive brain stimulation outcomes, these results also remind us of the importance of selecting an appropriate experimental design to probe the relationship between tDCS and modulation of behavior.

### Methodological differences between studies

For the reasons outlined in the section Rationale for Procedural Differences between Experiments, the experimental design was modified in this conceptual replication relative to Benwell et al. (2015) with the aim of simplifying the study parameters. In particular, the reduction of the stimulation duration from 20 to 15 min could have influenced the results, however the significant effects in Benwell et al. were visible within the first 15 min of stimulation, and were also evident after just 10 min of bi-parietal stimulation in Giglia et al. (2011). Therefore we still expected to see an *intensity* × *performance* interaction using this design, if it were present.

Secondly, the increased duration of each experimental block (3–4 min in Benwell et al. vs. 7 min here) might have led to increased fatigue in our participants, caused by a higher demand on sustained attention resources. In spatial attention paradigms, decreased arousal/alertness (often driven by increased time-on-task) typically generates a reliable left-to-right shift of spatial bias (Bellgrove et al., 2004; Fimm et al., 2006; Dufour et al., 2007; Dodds et al., 2008; Benwell et al., 2013a,b; Newman et al., 2013, Benwell et al., 2017; Veniero et al., 2017). This is likely to be due to depletion of right-hemisphere lateralised ventral (sustained) attention resources which, in turn, leads to a disruption of the right dorsal (spatial) attention network (Corbetta and Shulman, 2002, 2011; Corbetta et al., 2005). However, there was no overall rightward shift of spatial bias evident here, making this explanation for the difference in outcomes unlikely.

### Non-linear and state-dependent effects of tDCS

Whilst this data may introduce some questions regarding the reliability of the non-linear, state-dependent effect of current strength in this particular sample of individuals, it is possible that an additional set of yet-unknown variables are responsible for the failure to replicate here. Due to the large parameter space of the tDCS technique, that has yet to be fully explored and documented (Fertonani and Miniussi, 2016), it remains possible that the two sample groups differed in still more complex ways. Although the participants in the present study and in Benwell et al. (2015) were similar in terms of age (mean age = 21.43 vs. 22.9, respectively) and gender (16F/13M vs. 19F/19M), there may have been additional differences in, for example, neurochemistry, caffeine intake, and menstrual phase, all of which are prone to influence the excitability of the target neuronal populations, rendering them more (or less) amenable to the effects of stimulation. Yet without these important pieces of the puzzle we find ourselves some way short of developing a reliable means of modulating cognition and behavior using this technique.

## Conclusion

We aimed to replicate our previous study (Benwell et al., 2015), where we found a non-linear, state-dependent interaction between tDCS intensity (*1mA vs. 2mA*) and baseline performance (*high- vs. low precision*) on a visuospatial attention task. A within-groups design (vs. between-groups in the previous study) identified no overall effects of tDCS on spatial bias, and no current × performance interaction. These results highlight the need for robust replication of positive tDCS results to better understand the efficacy of, and mechanisms involved in, non-invasive brain stimulation.

## Author contributions

GL conceived the study. GL, FF, NS, MC, and GM collected the data. GL, FF and NS analyzed and interpreted the data. All authors wrote the manuscript.

### Conflict of interest statement

The authors declare that the research was conducted in the absence of any commercial or financial relationships that could be construed as a potential conflict of interest.
